# An Analytical Review of the Structural Features of Pentatricopeptide Repeats: Strategic Amino Acids, Repeat Arrangements and Superhelical Architecture

**DOI:** 10.3390/ijms22105407

**Published:** 2021-05-20

**Authors:** Sailen Barik

**Affiliations:** EonBio, 3780 Pelham Drive, Mobile, AL 36619, USA; eonbiohelp@gmail.com

**Keywords:** protein structure, tricopeptide repeats, PPR, helix, protein-RNA interaction, solvation

## Abstract

Tricopeptide repeats are common in natural proteins, and are exemplified by 34- and 35-residue repeats, known respectively as tetratricopeptide repeats (TPRs) and pentatricopeptide repeats (PPRs). In both classes, each repeat unit forms an antiparallel bihelical structure, so that multiple such units in a polypeptide are arranged in a parallel fashion. The primary structures of the motifs are nonidentical, but amino acids of similar properties occur in strategic positions. The focus of the present work was on PPR, but TPR, its better-studied cousin, is often included for comparison. The analyses revealed that critical amino acids, namely Gly, Pro, Ala and Trp, were placed at distinct locations in the higher order structure of PPR domains. While most TPRs occur in repeats of three, the PPRs exhibited a much greater diversity in repeat numbers, from 1 to 30 or more, separated by spacers of various sequences and lengths. Studies of PPR strings in proteins showed that the majority of PPR units are single, and that the longer tandems (i.e., without space in between) occurred in decreasing order. The multi-PPR domains also formed superhelical vortices, likely governed by interhelical angles rather than the spacers. These findings should be useful in designing and understanding the PPR domains.

## 1. Introduction

**Figure 1:**
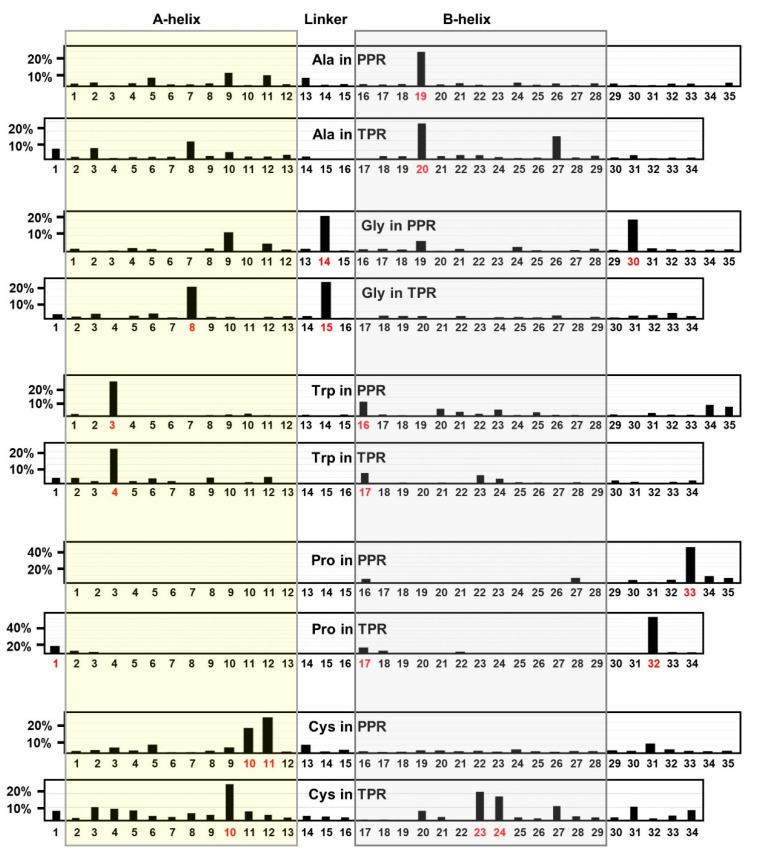
Positional distribution of individual amino acids over penta- and tetra-tricopeptide repeats. Naturally occurring tetra- and penta-tricopeptide repeat sequences (a total of 22,999 PPRs and 1137 TPRs) retrieved as described previously [10] and in the Materials and Methods. For each amino acid, the total number in each position of the repeat is counted using Excel, and plotted as the percentage of the total number of that amino acid in all positions (35 in PPR and 34 in TPR). For example, the percentage of Ala at any PPR position is = (Na/Nt) × 100, where Na is the number of Ala at a given position a in all PPRs, and Nt is total number of Ala in all positions in all PPRs. Note the Y-axis scale for Pro is higher because the bulk of it (~50%) is present at a single position (as Pro33/Pro32), which in fact serves as a signature for these repeats [2,3,4,10]. Numbers in red on the X-axis indicate the major site(s) of concentration of a residue. A-helix and B-helix are boxed in transparent yellow and grey, respectively, with the linker sequence in the middle.

The α-helical repeats, represented by TPR (tetratricopeptide) and PPR (pentatricopeptide), consist of 34- and 35-amino acid units, respectively [1]. Each unit contains two helices that are 10–15 aa long and generally designated A and B (Figure 1). When multiple repeats occur in a polypeptide, they are connected by linkers of a range of lengths. In recent years, we and others have analyzed and presented various features of their sequence, higher order structure, and the arrangement and spacing of the repeat motifs [2,3,4,5,6,7,8,9,10]. Being the better studied member of this family, TPR has been compared and contrasted with PPR [4,9,10,11,12]. Notwithstanding these studies, the molecular details of the structure of both repeats, particularly PPR, have remained incomplete. Pioneering research in the early days of protein structure provided a detailed architecture of the two major structural elements of a polypeptide, namely the α-helix and β-strand (sheet), including helical pitch, angle, and positional amino acid preferences [13,14,15,16,17,18,19,20]. Comparable studies have been recently conducted on TPR, specifically on rationally-designed three-TPR modules (designated as 3-TPR), which represent the most abundant type of TPR pattern in nature [2,11]. Such studies have been aided by experimentally determined three-dimensional (3D) structure of a few TPR domains [21,22,23,24,25], including the recently published crystal structures of interferon-stimulated gene 58 (ISG58) [23,26], also known as IFIT5 (since it is a member of the IFIT–interferon-induced protein with tetratricopeptide repeats–family that comprises three other IFIT proteins). Unlike most other TPR protein structures solved, ISG58 is an essentially all-helical protein with a total of 24 helices, of which ~11 are tandem TPRs and the others are TPR-like [26,27]. PPR domains appear to be much more abundant in nature, since each plant genome encodes 400–600 proteins of this family and novel sequences continue to appear through massive gene expansion [28,29]. PPR domains are primarily involved in sequence-specific RNA binding and typically bind single-stranded RNA. Thus, many PPR-containing proteins are involved in RNA metabolism and RNA-related functions, such as splicing, translation, RNA editing and processing [3,4,5,6,7,8]. TPR, in contrast, participates in protein-protein interaction [1].

The goal of this bioinformatic study (in part) was to harness the advanced knowledge of TPR structures to understand the higher order structure of the PPR domains which often occur in large congregations of multiple repeats, reminiscent of animal ISG58. This should bring to light the structural determinants and properties of the architecture of pentatricopeptide repeat domains.

## 2. Results

### 2.1. Location of Key Amino Acids in TPR and PPR; a Comparative Analysis

It is now well-established that the tricopeptide repeats display a consensus pattern of length and several signature amino acid residues at specific positions, which were identified by the use of logo plots in previous studies [4,10,11]. The pattern is similar in TPR and PPR, although the residues are shifted by one position; thus, in TPR/PPR position numbers, the three most prominent signature residues, written in single-letter amino acid codes, are: G15/14, A20/19, and P32/33. It is likely that the positional shifts have implications in the specificity of their function. In contrast to TPRs, which are consistently 34-amino acid (aa) long, the PPRs make up a diverse family that includes not only the canonical 35-aa long founding member but also larger and shorter versions that contain several PPR signature residues [4,5,6,7,8,9,10,11,12,13,14,15,16,17,18,19,20,21,22,23,24,25,26,27,28,29,30]. Moreover, consideration of the spacer (linker) sequences as continuation of the adjacent PPR can create the impression of larger PPR-like motifs [10]. Together, this diversity makes it difficult to define the conserved amino acid residues in all PPRs. For this reason, I have focused on canonical 35-aa PPR and 34-aa TPR motifs and compared the patterns.

This study also marked a shift away from the traditionally used logo plot, since a logo plot identifies amino acids that dominate a particular position over other amino acids, but does not show the distribution of a particular amino acid over the entire length of the sequence. For example, although Pro was clearly shown to be favored at residue 32 and 33, respectively in TPR and PPR, over all other amino acids, it might share other positions with multiple amino acids, in which case its logo height would be reduced by dilution. Nonetheless, it could play important roles in those positions as well. Here, several patterns of location have been reported for selected amino acids and assessed in terms of their properties and contributions to protein structure and function.

#### 2.1.1. Alanine Locations in TPR and PPR

Ala20 deserves special mention as a major landmark residue in the TPR consensus [2,31], and as shown in the positional survey (Figure 1), A20 of TPR and its equivalent A19 of PPR are also most abundant in these positions.

**Figure 2:**
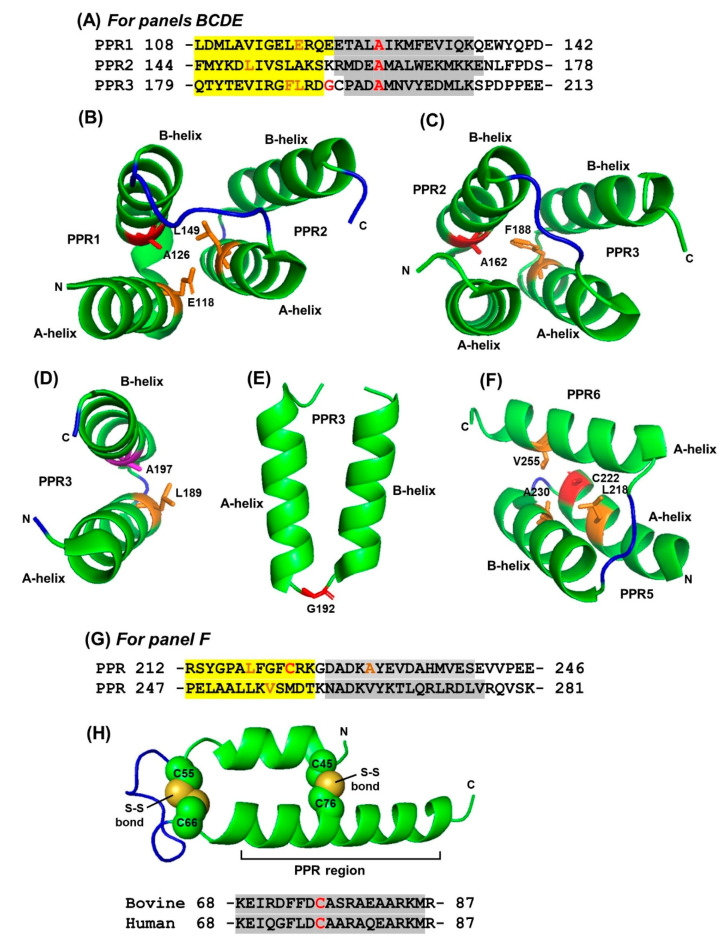
Interaction between the side chains of selective amino acid residues in pentatricopeptide repeats (PPRs). The 3D structures displayed by PyMol in panels (**B**–**F**,**H**) correspond to the amino acid sequences in panels (**A**,**G**,**H**), as labeled (details in Results). A-helix and B-helix sequences are shaded yellow and grey, respectively. The sequence in panel (**A**) is a set of three-PPR repeats in the *Arabidopsis thaliana* ‘thylakoid assembly 8-like protein’ found in the chloroplast (GenBank Q9STF9; PDB 4LEU). In each repeat, the invariant A20 residue and a glycine are in red color, and the major residues that the interact with are in orange, as shown in the panels (**B**–**E**). Panel (**G**) shows two tandem PPRs in proteinaceous Ribonuclease P1, also from a six-PPR domain in *Arabidopsis thaliana* chloroplast (Q66GI4), corresponding to the structure in panel (**F**) (PDB 4G24). Panel (**H**) shows the part of a unique CHCH/PPR domain conserved in bovine and human, which is involved in two pairs of intramolecular Cys-Cys disulfide bonds as shown (details in Results, Section 2.1.5).

Nonetheless, Ala is spread over the full length of the repeat motifs, although it also displays higher occurrence at TPR positions 8 and 27, both of which are located in the helices (Figure 1 and Figure 2). The short nonpolar side chain of Ala in the TPR allowed hydrophobic interactions of a helix, and may also serve a similar role in the PPR helices. Two representative examples from a 3-PPR protein are shown here (Figure 2A), in which the conserved Ala in two PPR units undergo stabilizing interactions with L149, E118 (Figure 2B), F188 (Figure 2C), and L189 (Figure 2D). Leu and Phe are hydrophobic residues and therefore, their side-chains interact with that of Ala by hydrophobic stabilization. Although Glu is an acidic residue overall, the hydrocarbon portion (-CH_2_-CH_2_-) of its side-chain is long enough to present a surface that interacts with Ala. Regarding location, note that A20 resides in the B-helix of a PPR (Figure 2B–D), and as shown, engages in stabilizing interactions that are either intra-PPR or inter-PPR; for example, A126 and E118 both are in PPR1, whereas L149 is in PPR2 (Figure 2B). A162 and F188 are also in two different PPRs, viz. PPR2 and PPR3, respectively (Figure 2C). In another interacting pair, both A197 and L189 are in PPR3 (Figure 2D). Thus, regardless of PPR distribution, these interactions always occur between residues in adjacent helices. This supports and extends previous findings, which showed that even a non-PPR helix can offer stabilizing interactions with a PPR helix, dictated mostly by hydrophobic interactions [32]. As yet, no specific role besides hydrophobic interaction has been assigned to Ala20/19 in the TPR/PPR.

#### 2.1.2. Glycine

Like Pro (See Section 2.1.4), Gly also tends to disrupt helices—though not as strongly as Pro, and for a different reason. The single hydrogen side chain makes glycine the smallest and also the only symmetrical amino acid with high conformational flexibility such that it is thermodynamically unfavorable for the highly constrained α-helix. As helix disruptors, both Pro and Gly are often considered “cap” residues that indicate the beginning (N-cap) and end (C-cap) of a helix. Lastly, because of their oddities, neither amino acid conforms to the typical Ramachandran plot. With a nonexistent side chain, Gly does not make major interactions with other residues, and is often found in flexible linker or loops, as exemplified by its location between the A-helix and B-helix of some PPRs (Figure 2D).

As shown previously [10], the G8 residue is exclusive to TPR and occurs in the middle of the A-helix, but is absent in the B-helix. G15, in contrast, occurs in both TPR and PPR, and is located in the linker between the two helices (Figure 1 and Figure 2E), perhaps acting as a C-cap helix-terminator for the A-helix in both. Gly30, on the other hand, may act as a C-cap residue for PPR only, but not for TPR. In contrast to PPR, the TPR contains a greater percentage of Gly, distributed over the length of the repeat.

#### 2.1.3. Tryptophan Locations in TPR and PPR

Previously, W4 was reported to be a consensus residue in the TPR motif [11], although the sequence logo plot revealed that this residue and the corresponding W3 in PPR are only weakly dominant [3,6,10]. The logo plot additionally revealed that two other aromatic acids, namely F and Y, may serve as conservative replacements, and as shown elsewhere [32], can be just as abundant in these locations. Tryptophan is not significantly dominant in any other position of either TPR or PPR (Figure 1), although some abundance is observed on the cusp of the B-helices (at the end of the connecting loop) of both repeats (Figure 1), where it may interact with ligands. The low overall abundance of tryptophan in the tricopeptide repeats is consistent with its rarity in the cell. Being aromatic in nature, F/Y share some of the properties of Trp; namely, strong hydrophobic interactions with other hydrophobic side chains at proper distance, such as with Ala, as shown earlier (Figure 2B).

#### 2.1.4. Proline Locations in TPR and PPR

Much has been written about P32/P33 in TPR/PPR [2,10,11]. Along with A20/19, it is the most conserved signature residue in both repeats. It was therefore used as a point of reference and the salient features of its location will be reviewed here. Proline (Pro) holds a unique place among the amino acids because the alpha-amino group is a secondary (imino) amine in Pro and not available for hydrogen-bonding. Pro, therefore, acts as a helix-breaker, not to be found in the interior of a helix. In rare occasions, when it does occur inside a helix, it produces a significant kink or bend, thus distorting the helix. For the same reason, Pro can be viewed as a natural helix terminator that ensures the end of a helix, which has been accepted as a universal rule. In fact, Pro in the helix is found in the N-terminal first turn of a helix (commonly as the 2nd residue of the helix), where the loss of the H-bond to the imino nitrogen does not have any significant effect [33,34]. Since the helix is the dominant structure in the tricopeptide repeats, I have placed special emphasis on these two amino acids and inspected their locations relative to the helices.

Each TPR/PPR unit is composed of two helices, generally designated as A-helix and B-helix, each ~13 residues long and separated by a spacer of ~3 residues [2,10]. Proline is an established helix breaker, which befits its absence within the helices and its presence at the end a repeat unit—specifically, at the end of the B-helix, i.e., position 32 in TPR and 33 in PPR (respectively denoted as 32@TPR and 33@PPR), which was previously shown by logo plots [10]. To determine if Pro may also occur in other positions, for example, at the end of the A-helix, the number of Pro at each position of TPR and PPR was counted and graphically plotted. The results (Figure 1) showed that Pro was clustered in three areas in the TPR: at the beginning of each helix (A and B, respectively 17% and 12% of total Pro), and, most frequently, ~3 residues after the B-helix (47% @32), which is the signature P32, mentioned earlier. Studies of synthetic tri-TPR peptides containing identical repeat units (referred as 3TPR) led to the suggestion that the P32 forces a turn to occur, thus terminating the helical structure between TPR motifs [2], which was also supported by evidence from naturally-occurring TPR [21,35]

**Figure 3:**
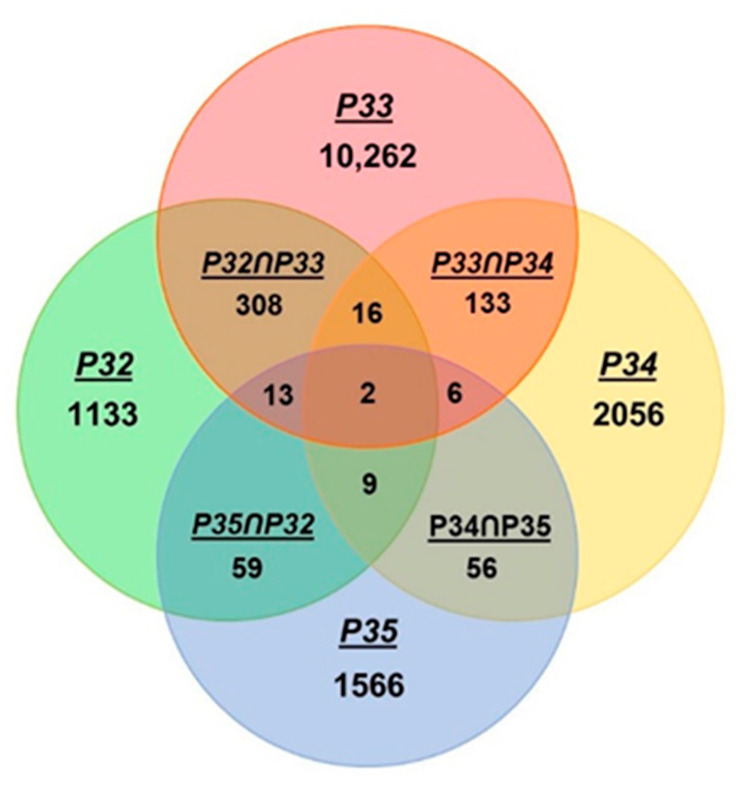
Combinatorial locations of Pro residues in PPR. The occurrence of Pro at the distal end of PPR was counted by Excel’s ‘countif’ function as described in Results (Section 2.1.4). Colocalizations of Pro at multiple sites were also counted similarly and the numbers presented in the overlapping areas of the Euler diagram (which may appear similar to the more commonly known ‘Venn diagram’).

The Pro residues in PPR, in contrast, resided in two areas: at the beginning of B-helix (8% @16,17) and at higher numbers spread over ~2–6 residues following the B-helix (69% @ 30–35) with the signature P33 at the center. Specifically, the Pro residue(s) near the beginning of the TPR A-helix was missing in the PPR. The known property of Pro as helix breaker attracted further attention to its extraordinary high concentration at the end of the PPR (Figure 1) and therefore, we explored its distribution pattern over the four positions, viz. 32, 33, 34, and 35 [10]. In this approach, the occurrence of adjacent Pro residues in 26333 PPR units was noted using Excel’s ‘countif’ formula and the results were plotted in a four-circle Euler diagram (Figure 3).

Results confirm that position 33 indeed takes the lion’s share of Pro in this area (10262), compared to the second highest number (2056) at 34, but a considerable number of di-Pro (PP) were also noted, as shown in the intersections. The most abundant were P32ՈP33 (308), followed by P33ՈP34 (133), evidently contributed by the common P33. A smaller number of tri-Pro (PPP) were found as well, and the most abundant ones contained P33 at the center, i.e., P32-33-34 (16). Proline quadruplet (P32-33-34-35) was practically nonexistent in PPR and occurred only twice in our 26333-strong cohort; they were the last two PPR units of a 16-PPR soybean protein (K7MEB5) and were essentially identical in sequence (34 out of 35 resides were identical). To conclude, Pro33 appears to be the most crucial helix-terminating residue in PPR, functionally equivalent to P23 of TPR, and the neighboring Pro residues likely played accessory roles to various extents.

#### 2.1.5. Cysteine

PPRs in our collection contain an average of about one Cys per PPR, which are distributed rather evenly over the PPR length, compared to, say, Pro (Section 2.1.4), although some concentration is seen at 10/11 in PPR/ TPR, and at 23, 24 in TPR (Figure 1), all of which are within helices. Due to this, the reversible Cys-Cys disulfide bond often assists a protein in attaining the natural fold, and in such cases, reductive destruction of the bond may lead to loss of the structure, and often times, function. However, Cys in PPRs were generally not engaged in disulfide bond formation (with one interesting exception, which is described later). An example, representative of the majority, is shown for the *Arabidopsis thaliana* proteinaceous ribonuclease P (RNase P), in which Cys222 of PPR5 (212–246) in the six-PPR domain interacts with three aliphatic residues (namely, L218, A230 and V255) (Figure 2F), clearly involving hydrophobic interactions without using the SH group of Cys. Two plant PPRs contained a run of four Cys (CCCC): K7MN15, an uncharacterized soybean (*Glycine max*) protein, and G7JWA8, a putative protein in the barrel clover or barrel medic (*Medicago truncatula*). Such high concentrations of sulfhydryl side groups may serve exclusive roles in structure and function that are currently unknown.

An interesting and unique exception was noted in a protein of the eukaryotic mitochondrial 28S ribosomal subunit that interacted with mitochondrial IF3. It contained a single highly conserved PPR motif, as predicted bioinformatically (Figure 2H), which included a single invariant Cys (residue 76). The amino acid sequence of this protein is highly conserved in all species; the bovine and human orthologs, for example, are 87% identical. As expected, their higher order structures are also essentially identical, as revealed in the 3D structures of the 28S subunit (bovine PDB 6NEQ; human PDB 6RW4). Interestingly, this area folds into a coiled-coil-helix-coiled-coil-helix domain [36,37]. Like PPR, this domain (abbreviated as CHCH domain) is characterized by antiparallel alpha-helices and conserved residues in specific positions that are different from the PPR signature. Thus, the single PPR in this ribosomal protein also conforms to the sequence requirements of a CHCH domain. Additionally, the 3D structures also revealed that this protein made contact with multiple other 28S ribosomal proteins but not with the rRNA in the 28S subunit. Lastly, as seen in the bovine protein (PDB 6NEQ), the aforementioned Cys76, located in the PPR, and three other upstream Cys residues together formed two disulfide bonds (Figure 2H). In contrast, the recombinant human protein, used to generate the crystals from which PDB 6RW4 was derived, was purified in the presence of 2-mercaptoethanol to prevent any aggregation, and as a result, its structure lacked disulfide bonds, but generated an otherwise identical structure.

The remaining amino acids showed a comparatively unremarkable distribution over the entire length of PPR (as well as TPR), and thus, are not presented here.

### 2.2. PPR Length and Clustering

As stated earlier, PPR-containing proteins bind RNA in a sequence-specific manner [3,4,5,6,7,8,38]. The 3D structures of PPR-RNA complexes revealed that multiple contact points are required in these interactions [5,6,7,39]. However, our PPR protein collection contained several PPR domains with just 2 or 3 repeats and a few single repeats, which prompted an examination of the overall number and distributions of PPR clusters to find any pattern. In continuation of my prior interest in PPR linkers [10], I first considered two classes of PPR: ones that are conjoined (i.e., in tandem, without any amino acid in between) and those separated on both sides by spacer amino acids. We will refer to these two classes as tandem-PPR and lone-PPR, respectively. To illustrate, the middle PPR (in bold) in the following sequence is a lone-PPR, and it also contains a dimer and a trimer tandem PPR string:

-8 aa-(PPR)(PPR)-4 aa-(PPR)-12 aa-(PPR)(PPR)(PPR)-5 aa--.

**Figure 4:**
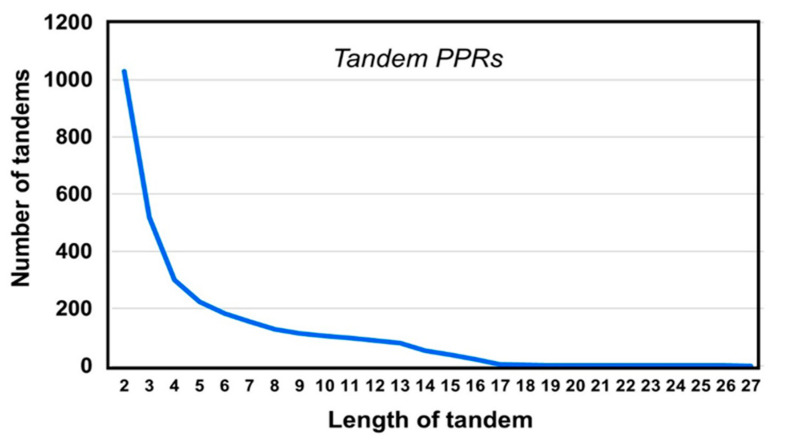
Arrangements of PPR. The graph presents the relative abundance of PPR clusters of different lengths. For example, 3 in the *X*-axis means a cluster of three tandem PPR units connected without any spacer amino acid between them, i.e., PPR-PPR-PPR. As seen in the graph, the PPR-PPR-PPR triplet was found 519 times in our collection of 2130 non-redundant PPR-containing proteins (details in Section 2.2).

The 2130 PPR proteins in our collection totaled 23,174 PPRs, averaging about 10 PPRs per protein. Of these, 3622 were lone-PPRs, averaging 1.7 per protein, which is equivalent to ~15% (3622/23,174 × 100) of total number of PPRs. Thus, the majority of PPRs (85%) were tandem-PPRs, i.e., they make contact with another PPR on one or both sides (Figure 4).

### 2.3. Interhelical Angles in PPR Domains and Superhelices

**Figure 5:**
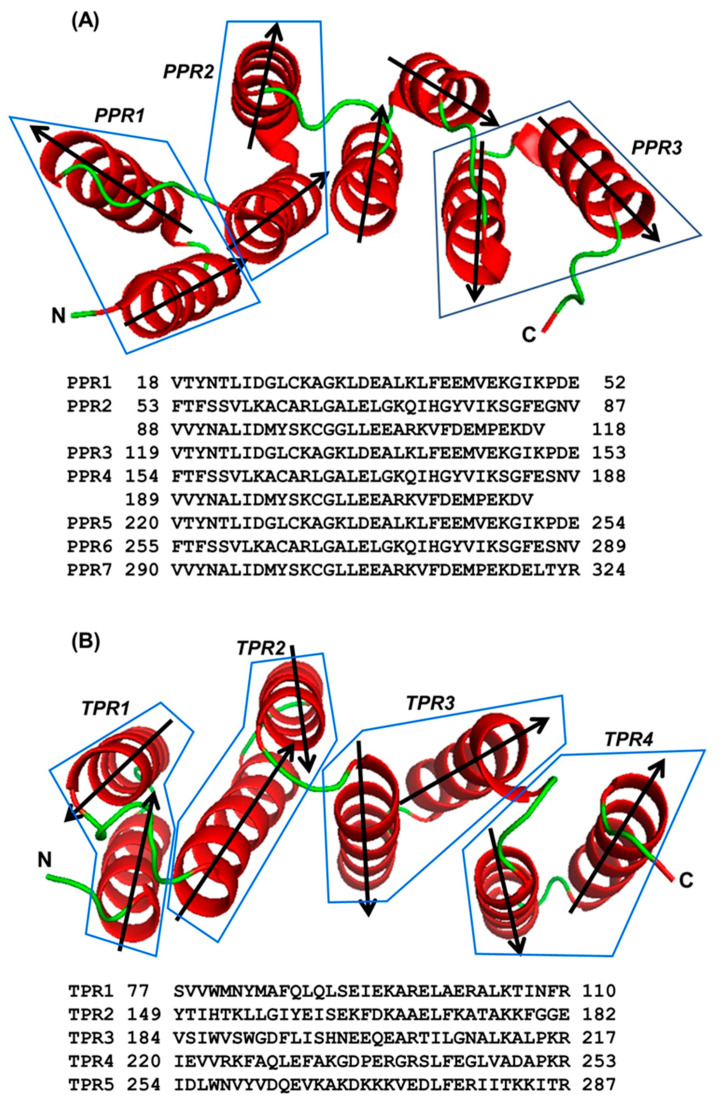
Superhelical structure of a multi-PPR domain. The superhelical structure of a PPR domain is shown along with that of a TPR domain to illustrate the similarities. (**A**) PPR domain of the plant ‘multiple organellar RNA editing factor’ (MORF, also known as ‘RNA editing factor interacting protein’ or RIP) from PDB 5IWW, Chain D [41]. The first three PPRs (out of a total of seven) are shown as representative, and demarcated by polygons. The two unboxed helices belong to a non-PPR sequence between the second and third PPR. (**B**) TPR domain (for comparison) of RRP5, an essential factor for ribosome maturation in yeast; PDB WWM, Chain B [24]. Only four TPRs of the multi-TPR C-terminal region are shown and each is demarcated by a polygon. In both panels A and B, the helices are dark red in color and the connecting linkers are green. The two termini of the peptide (amino, N and carboxy, C) are labeled, and the directionality of each helix is also indicated by arrowheads, which shows their antiparallel arrangement.

Structural analyses of the natural PPRs [10,30] and synthetic 3TPRs mentioned earlier [11] showed that the neighboring helices, both within and between repeats, contacted each other. In PPR, this is governed by proximity as well as noncovalent interactions, and the net energy that determines the final structure is the result of thermodynamically stabilizing and destabilizing forces of interaction between amino acid side chains [9], also supported by covariance analysis [2,40]. The aforementioned studies of synthetic 3TPRs (as well as several naturally-occurring multi-TPR domains) further revealed that the series of antiparallel helices formed a higher order of parallel array, generating a superhelical architecture. This has been likened to a spiral staircase, where each TPR motif is a step [11]. In IFIT5, the convoluted intramolecular packing of eight TPRs has been called TPR eddy for its vortex appearance [23]. Superhelices were also discernible in recent analysis of naturally-occurring PPR domains [10], an example of which is shown (Figure 5). In fact, the superhelical architecture allowed proper contact and interaction between specific amino acid residues of the PPR and specific of RNA, the most common functional ligand of PPR, leading to the proposition of an RNA recognition code in the PPR sequence [5,6,7]. In this interaction, each RNA nucleotide is coordinated by two amino acid residues in each PPR unit, such as aa4 and aa34 [3,10]. Thus, replacement of one of these amino acids may alter the recognition specificity, causing a shift to binding a different RNA sequence. For instance, a Thr4-Asn34 duo would bind the nucleotide Adenine, whereas a Asn4-Asp34 duo would bind Uridine instead [6,7]. This arrangement also offers a one-to-one relationship of the primary structure of the PPR domain and the RNA, such that an 8-PPR superhelix would bind a specific 8-nucleotide long RNA sequence [3].

As simple as this model is, the superhelical fold is found in both ligand-bound and unbound crystals (i.e., co-crystal and Apo-crystal) of a PPR; thus, it is unclear what role, if any, the ligand plays in stabilizing the superhelix. Overall, what dictates the superhelical architecture and whether the tricopeptide amino acid signatures play a role in its formation has remained unresolved, a situation which I intended to revisit. To start with, I retrieved the experimentally determined X-ray crystal structures of multiple PPR (and TPR) proteins and measured the angles between the helices using PyMol, as described in Materials and Methods (Section 4.2). Since the superhelicity of the full domain was to be analyzed, it was necessary to be able to directly compare all types of inter-helical angles. The intra-PPR angle was between A1 and B1 (denoted as A1ΩB1) and the inter-PPR angle that follows was B1ΩA2, and so forth.

**Figure 6:**
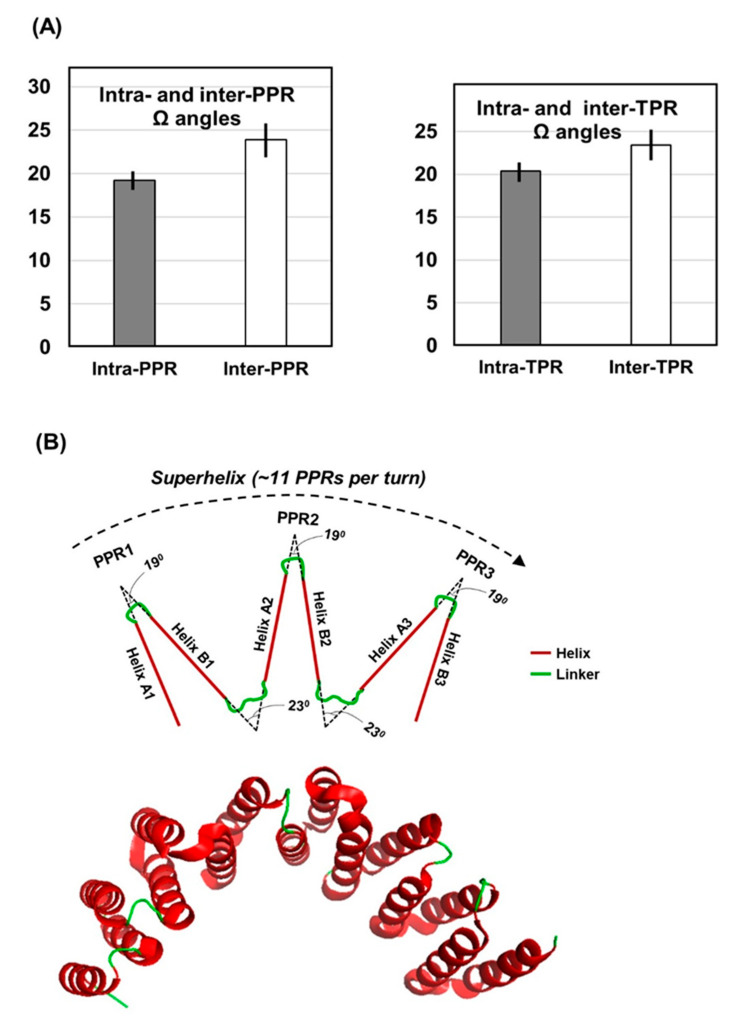
Helical angles in PPR domains. (**A**) Interhelical angles (Ω) determined from PDB crystal structures; the averages of 208 intra-PPR, 234 inter-PPR, and 216 each of intra- and inter-TPR helical angles are shown. The exact values and the standard errors are in parenthesis: intra-PPR (19.22 ± 1.37), inter-PPR (23.9 ± 3.03); intra-TPR (20.36 ± 1.63), inter-TPR (23.41 ± 3.27). (**B**) Schematic of location of the inter-and inter-repeat helical angles are located in the context of a superhelix, which was found to contain approximately 11 PPRs per complete turn. For a realistic perspective, an actual superhelix of a multi-TPR domain (PDB 5C9S, a portion of which was shown in Figure 5B) is shown below. Note that the superhelices of PPR and TPR are virtually identical.

The measurements are presented here, along with a schematic drawing (Figure 6A,B). As the 3D structures of long PPR domains have not been solved, I extrapolated the geometry of the shorter domain (Figure 6B) and estimated that the complete turn of a PPR superhelix consisted of roughly 11 PPR units. The observations can be summarized as follows: (a) Angles in each class (intra- and inter-repeat) fell within a narrow range of values, suggesting a conserved superhelix structure; (b) Intra-repeat angles (A1ΩB1) were significantly smaller than the inter-repeat ones (B1ΩA2) (i.e., 19.220 vs. 23.90 in PPR; 20.360 vs. 23.420 in TPR), indicating that each repeat unit (e.g., A1B1) is a tight structure, whereas two adjacent repeats are separated by a wider angle. (c) An extensive study of linker sequences connecting the PPR units showed that the larger ones often formed bihelical regions [10]. In fact, their 3D structures were indistinguishable from those of the true PPRs, such that they did not interrupt the continuity of the PPR superhelix (Figure 5A). This was true regardless of their location with respect to the PPRs, i.e., whether inserted between two PPR units or flanking a PPR domain on either side. Preservation of the superhelical vortex by these imposter PPR units has been confirmed by the measurement of the helical angles, i.e., they displayed PPR-like angular values within the unit and with the neighboring true PPRs (data not shown). It is fair to infer that the superhelices are resilient to diverse linkers and maintain their stability and architecture regardless of linker sequence or length [12,42,43].

### 2.4. Regulation of Repeat Folding by Interaction between Neighboring Helices

The results presented above are in accord with previous thermodynamic analyses showing that non-TPR helices downstream of a TPR domain can provide net energy of stabilization for the TPR through side-chain interactions, complying with the same molecular principles that operate between TPR helices [9,11,42].

**Figure 7:**
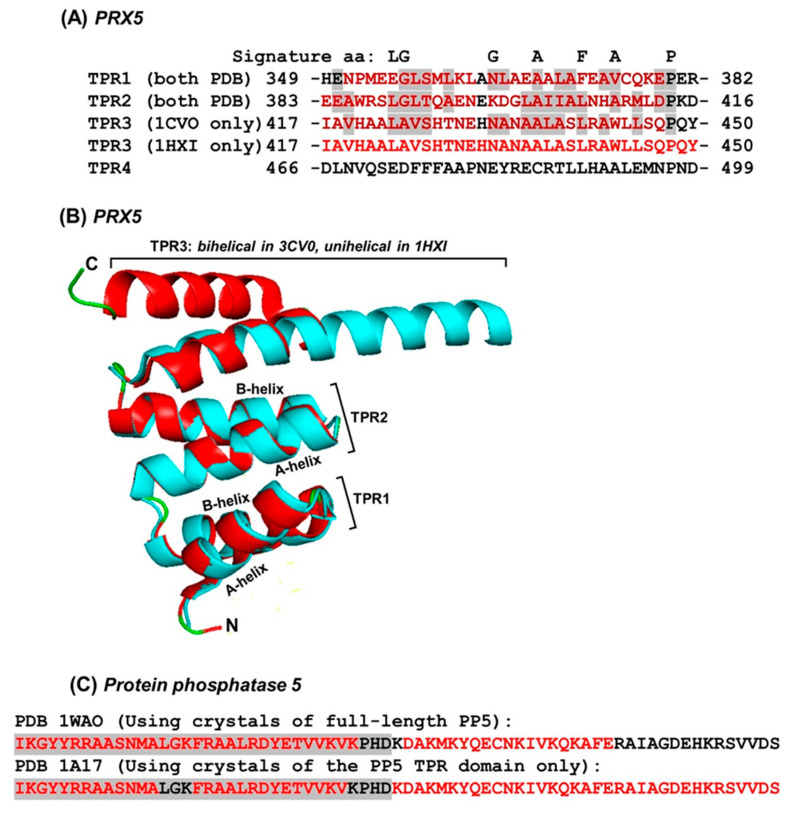
Single-helix (unihelical) TPR units. (**A**) Only the first four TPR sequences of the seven-TPR *T. brucei* PRX5 protein are aligned to mark the similar amino acids in TPR1, TPR2 and one TPR3 (shaded in grey, if present in two or more TPRs) while the general TPR signature residues are shown on top. The A-helix and the B-helix are colored red; note that the two helices of TPR3 are discernible in the structure of the full-length protein that contain all seven TPRs (PDB structure 3CV0), but form one continuous long helix in the truncated protein (PDB structure 1HXI), indicated by the continuous red font color. (**B**) The first three TPR units of the 3CV0 (red color) and 1HXI (cyan color), corresponding to the sequences in Panel A, are superimposed using the merge function of PyMol. Note the bihelical structure of TPR3 in 3CV0 (red), and unihelical structure of the same TPR in 1HXI (cyan); TPR1 and TPR2 have retained the same bihelical structures in both constructs, as seen by complete superimposition. (**C**) Comparison of the helical regions, observed by X-ray diffraction of crystals of the full-length protein phosphatase 5 (PP5) and those of the 3-TPR domain only, devoid of the downstream sequence (PDB 1WAO and 1A17, respectively). Only the TPR3 (shaded grey) and the relevant portion of the downstream sequence are shown, as TPR1 and TPR2 structures were very similar in the two crystals. All helices—TPR and non-TPR—are colored red. Note that the shorter fragment of PP5 has the canonical bihelical architecture, whereby the LGK loop region separates the A- and B-helices (not marked); the TPR is followed by a long 35-aa helix (KDA…VDS). In contrast, the full-length protein (1WAO) shows a unihelical structure of the TPR, followed by a shorter helix, only 19-aa long (DAK..AFE).

A fortuitous structural finding of a truncated TPR domain lent further credence to this mechanism, adding new dimensions to our knowledge of tricopeptide architecture. The crystal structure (PDB 3CV0) of the seven-TPR C-terminal domain of *Trypanosoma brucei* peroxisome targeting signal 1 receptor PRX5 [44] exhibited the canonical A- and B- helices of each TPR (Figure 7A). Unexpectedly, when the crystal structure of a truncated fragment of the same polypeptide (PDB 1HXI), containing only the first three TPRs, was determined [35], only the first two TPR units was found to possess canonical bihelical structures whereas the third TPR was a single 34-aa long α-helix (Figure 7B). In other words, the hairpin configuration, in which the two-helices of TPR3 were connected by a linker at the bend, opened up to generate a single continuous helix [12,35]. The primary structure of this novel unihelical TPR3 did not reveal anything remarkably different from the others that could explain the continuous helix (Figure 7B). However, since the continuous helix was coincidental to the loss of the downstream TPR, I examined the interactions between these two TPRs in the full-length protein, as described before [9]. Addition of the major side chain interaction energies (>1 in magnitude) (Table 1) showed that the TPR4 attracts with the TPR3 B-helix with a robust force of 150.88 kJ/mol, which would surely facilitate stabilization of the TPR3 B-helix in its canonical location in the domain architecture.

These results reiterate the important role that helix-helix interaction plays in the creation of the superhelical vortex in TPR and PPR [9,12]. Although systematic helix deletion analyses have not been conducted in either family, a few instances are known in which the helical architecture is rearranged in shorter fragments of a repeat or under different physical conditions [12,44]. In one of the earliest examples, structural studies of the Ser-Thr protein phosphatase 5 (PP5) revealed that the helical architectures of the last TPR and its downstream sequence were different between the crystals of the full-length protein [45] and those of the TPR-only polypeptide fragment [31]. As shown in detail (Figure 7), the helix lengths and boundaries were rearranged in the two structures. Although the exact reasons for this are unclear, the ability of the TPRs to morph into multiple structures fits their role in protein-protein interaction, which often calls for flexibility and structural switch. Specifically, in PP5, the TPR domain folds to autoinhibit the catalytic channel, which is relieved in part by its interaction with heat-shock protein 90 and long-chain fatty acids, important for physiological regulation of PP5 function [45,46]. In a different example, bovine cyclophilin 40 (CyP40) was found to form crystals of two different geometries (monoclinic and tetragonal) under two different conditions of crystal growth [21]. Interestingly, the two crystal forms also showed dramatically different conformations of the molecule. In the monoclinic form, the three repeats of the TPR domain comprised a total of seven helices of variable length, whereas in the tetragonal form, two of the helices straightened out to form a single extended helix. As with PP5, this had important effects on the TPR domain, relevant to its ability to bind Hsp90 and calmodulin, the two interacting partners of CyP40 [21].

## 3. Conclusions, Discussion, and Future Directions

In previous studies [10] and those described here, visual inspection and interhelical angle measurements revealed no appreciable differences in the superhelical structures of purely PPR domains and those interspersed with non-PPR bihelical sequences. We can, therefore, conclude that the formation of the superhelical structure per se does not require the PPR motif or its signature residues, and that any bihelical unit may suffice. However, it is difficult to predict whether an exclusively non-PPR repeat, consisting of highly diverse helices, will also form a stable superhelix of identical dimension. In fact, diverse types of repeats form superhelical architectures of different dimensions, built with tandem arrays of α-helix or β-strand or a combination of the two, which allows specific ligand binding [2,47,48]. For interested readers, a few examples of the 3D structures are: Leu-rich repeat (PDB 1LRV), ankyrin repeat (1AWC, 1IHB, 1LRV), and WD repeat (1YFQ). In all cases, key residues play direct roles in functionality, and the ‘RNA recognition code’ described earlier for PPR (Section 2.3) implies that a non-PPR superhelix is likely to be defective in RNA binding, or bind a noncognate RNA sequence of questionable function [5,6,7]. The spacing between the PPR repeats in a protein continues to be intriguing, since the length and composition of these linkers show little or no similarity in either length or sequence [10]. As the superhelicity is conserved, it is imperative that the linkers exert little influence on the architecture of the PPR domain. To state it from another perspective, the superhelical regions around tandem PPRs and lone PPRs (i.e., demarcated by linkers) are indistinguishable in the 3D architecture.

Notwithstanding the helix-terminating role of proline, many PPRs were devoid of Pro; a few examples can be found in UniProt# A0A0R0H859, A0A0R0GQA3, K7K2X4, A0A0R0JRI1. It is possible that helix termination in some of these PPRs was facilitated by Gly, such as G30, but in others, it could have been due to the lack of helix-forming residues. As mentioned for the multi-structural TPRs (Section 2.4), the architecture of a tricopeptide repeat is not dictated solely by the intra-repeat nonhelical residues or the inter-repeat linkers, but also by complex interactions involving multiple side chains and solvent conditions.

The PPR family also contained several examples of nearly identical PPR motifs and domains, suggesting domain shuffling through gene duplication events. As previously noted, such duplicated domains could be a few hundred amino acids long and found in different species of plants [10]. For example, UniProt# K7M927 and A0A072TSC5, two PPR proteins encoded in the genome of the same two plants mentioned earlier (soybean and barrelclover, respectively), both had a 422-aa long 12-PPR domain with 87% amino acid identity (99.2% similar if conservative replacements are included). The abundance of near-identical repeats in PPR may be due in part to the vastness of the plant kingdom; the flowering plants (Angiosperms), for example, consist of about 300,000 known species, and the number may be increasing [49]. The evolutionary mechanism of such duplications has not been pursued. It is also not known whether or not these orthologs have nonredundant biological functions.

In a variation of this theme, multiple PPR entries were found that had overlapping PPR domains, sharing only a subset of the repeats, but not others. The likely scenario of two isoforms of a PPR protein with different number of the repeats, which may have distinct nonredundant roles, is an exciting area of study. The smaller protein may encode a subset of the functions of the larger isoform, a situation comparable to alternative splicing [50]. ASmong other possibilities, it may also act as a dominant negative inhibitor. In such cases, I have only analyzed the longest protein, in order to conserve space, but a few random pairs in the UniProt collection are listed here: A0A396JIR4 and A0A072VRD3; G7L8W9 and I3SE20; I3T018 and G7IAS0; G7L4F1 and A0A396HSB0; G7IHD2 and A0A396JG33; A0A072VJT0 and I3RZB5; G7L321 (12 PPRs) and A2Q3R3 (very small protein, contains the last PPR only); G7KLL8 (13 PPRs) and I3SWC5 (9 PPRs, missing 2 PPRs in front, 2 in the back). It is an interesting question whether orthologs in two different species exhibit such PPR shuffling. For example, a preliminary search detected the following two orthologs in human and mouse, respectively: P42704 (human), Q6PB66 (mouse). While their shared PPRs were identical, the linker lengths between them were different; moreover, the mouse protein had two extra PPRs. It is clear that systematic deletion, insertion and mutational analysis of the PPR and linker sequences with structural and functional analyses will shed light on their specific roles as well as their evolutionary history.

Finally, it is tempting to note that the longest PPR domain so far was found in the mitochondrial ‘PPR-containing protein 5 (PPR5)’ (Uniprot# Q10451) of fission yeast (*Schizosaccharomyces pombe*). It consisted of 25 PPRs spanning over 1036 amino acid residues and is likely involved in mitochondrial protein synthesis and mitochondrial biogenesis [51]. Structures of TPR or PPR domains containing large number of repeats have not been determined, which remains a challenge for the future.

## 4. Materials and Methods

The general methods of sequence retrieval and analysis have been described before [9,10] but will be briefly presented here along with specific procedures used in this study.

### 4.1. Retrieval of Sequences

Protein sequences were retrieved as described before [10], primarily from UniProt (https://www.uniprot.org/, accessed on 1 March 2021), but also from NCBI (www.ncbi.nlm.nih.gov, accessed on 1 March 2021). X-ray crystal structures of TRP and PPR were downloaded as PDB files from RCSB (http://www.rcsb.org/, accessed on 1 March 2021), and examined by PyMol [52], which also displayed the secondary structural elements (α-helix, β-strand, loop).

### 4.2. Analysis of Sequence Patterns and Interhelical Angles

When needed, TPR and PPR repeats were identified, mainly via the TPRpred website (https://toolkit.tuebingen.mpg.de/#/tools/tprpred, accessed on 2 March 2021), and verified by the descriptions in the corresponding entries in UniProt and NCBI, as previously described. The lengths and sequences of the interhelical and inter-repeat spacers were determined by visual inspection, and the lengths were recorded in Excel spreadsheets for subsequent analysis. PPR or TPRs containing specific amino acid residues at various positions in the repeats were programmatically filtered, and their numbers counted. A PyMOL script was written that used the anglebetweenhelices function to calculate the angle between two neighboring helices in experimentally determined 3D structures.

## Figures and Tables

**Table 1:** Energy of side chain interactions between TPR4 and the B-helix of TPR3 in PDB 1HXI.

TPR3 B-Helix aa number	Interacting aa in TPR4	Interaction Energy (kJ/mol)
Ala 433	Ala 477	−1.58
–	Phe 475	−1.09
Asn 434	Ala 478	−3.3
Leu 437	Tyr 482	−5.7
–	Glu 481	−5.33
–	Cys 485	−5.08
–	Phe 475	−3.88
–	Ala 478	−1.68
Leu 440	Cys 485	−2.87
–	Leu 489	−2.63
Arg441	Glu 484	−60.92
–	Leu 488	−3.47
–	Cys 485	−3.26
–	Glu 481	−2.47
Trp 443	Leu 489	−3.2
Leu 444	Leu 489	−4.06
Leu 445	Leu 488	−5.83
–	Glu 484	−1.04
Gln 447	Asp 499	−2.04
Gln 449	Asp499	−1.23
Tyr 450	Asn 496	−23.44
–	Ala 492	−4.18
–	Met 495	−2.6

Free energies of interaction determined at the web server described previously [9] (http://bioinfo.uochb.cas.cz/INTAA/, accessed on 8 March 2021). The major interacting amino acids (out of a total 33 interactions) are listed. The TPR3 B-helix and TPR4 span amino acid (aa) residues 433–450 and 466–499, respectively. Note that a negative interaction energy value indicates a thermodynamically stabilizing interaction.

## Data Availability

Not applicable.
